# *In Vitro* Antibacterial and Time-Kill Evaluation of the *Erythrina caffra* Thunb. Extract against Bacteria Associated with Diarrhoea

**DOI:** 10.1100/2012/738314

**Published:** 2012-11-11

**Authors:** Olufunmiso Olusola Olajuyigbe, Anthony Jide Afolayan

**Affiliations:** Phytomedicine Research Centre, Department of Botany, University of Fort Hare, Alice 5700, South Africa

## Abstract

The antibacterial activities of stem bark ethanolic extract of *Erythrina caffra* Thunb. against bacteria in diarrhoea was determined *in vitro* by the agar diffusion and dilution, macrobroth dilution, and time-kill assay methods. The result showed that the extract produced inhibition zones ranging between 15 ± 1.0 mm and 23 ± 1.0 mm, and the bacteria were susceptible at concentrations ranging between ≤100 and ≤1000 **μ**g/mL. While the MICs of the extract ranged between 39.1 and 625 **μ**g/mL, and the MBCs ranged between 78.1 and 625 **μ**g/mL, the MICs of *Micrococcus luteus*, *Proteus vulgaris* CSIR 0030, *Enterococcus faecalis* KZN, and *Staphylococcus aureus* OK_3_ were less than 100 **μ**g/mL, and the mechanisms of antibiosis indicated that the crude ethanolic extract was highly bactericidal against the entire test bacteria isolates. In the time-kill assay, the average log reduction of the viable cell count ranged between 0.916log _10_ and 1.851log _10_ cfu/mL on incubating the bacteria for 4 h at the MICs, while the reduction ranged between 0.183log _10_ and 1.105log _10_ cfu/mL after 8 h of incubation. Incubating the bacteria for 4 h at 2 × MICs resulted in the reduction of the viable cell count to between −0.264log _10_ and 0.961log _10_ cfu/mL, while the average log reduction ranged between −3.968log _10_ and −0.425log _10_ cfu/mL after 8 h of incubation with *Micrococcus luteus*, *Proteus vulgaris* CSIR 0030, and *Staphylococcus aureus* OK_3_ being the most highly affected bacteria. The result showed that the extract exhibited broader-spectrum antibacterial activity and justifies the use of *Erythrina caffra* in the folkloric medicine for treating gastrointestinal infections in South Africa.

## 1. Introduction

In medical parlance, antibiotic resistance defines the epidemiology and pharmacological therapy of infectious diseases while the increasing incidence of resistance to antimicrobials is a growing concern of the medical arena [1, 2]. To avert the menace of the pathogens resistant to antibacterial agents, attention has been focused on discovering new antimicrobial compounds of microbial and plant origins [3, 4]. Natural products, especially from plants, have been considered interesting alternatives for treatment [5] because they are rich in a numerous variety of secondary metabolites with antimicrobial properties [6]. These secondary metabolites which are pharmacologically bioactive compounds include alkaloids, flavanoids, tannins, anthraquinones, and phenolic compounds [7, 8].

Diarrhoea, an important clinical problem [9] and a leading cause of morbidity and mortality in developed and developing countries [10, 11], is an intestinal infection responsible for death in the elderly in industrialized countries [12] and the deaths of 3-4 million infants and young children each year worldwide [13]. It accounted for 1.78 million deaths in low- and middle-income countries [14]. Being a divergence from the established rhythm of bowel movement, it is characterized by an increase in frequency and fluidity of stools resulting from dysentery, food poisoning, bacterial and viral infections, allergy to certain foods, and changes in climatic conditions. Epidemiologically, rotavirus is the most common cause of mortality in children [15]. Important bacterial pathogens mostly implicated in diarrhoea are diarrheagenic *Escherichia coli, Salmonella typhi*, *Bacillus cereus*, *Campylobacter jejuni*, *Aeromonas hydrophila, Shigella *spp.*, Yersinia *spp.and *Vibrio cholera,* and the main causative parasites in contaminated domestic water supplies included *Giardia intestinalis* and *Cryptosporidium parvum* [16]. During oral rehydration, breast and complementary feeding in children is essential in reducing the discomfort and inconvenience of frequent bowel movements [17], and use of medicines derived from plants is sought after by the indigenous populace from traditional healers because they are cheaper than modern medicine [18]. Based on this concept, several studies have evaluated the effectiveness of some traditional medicines in treating diarrhoea on all the different continents [19], while a programme involving the use of herbal medicines for diarrhoea control in the developing countries was introduced by WHO [20].

*Erythrina* is a genus of flowering plants in the pea family, Fabaceae, consisting of nine different species including the *Erythrina lysistemon* and *Erythrina caffra *being common in the Southern Africa. They are often cultivated as ornamental, street, and park trees in drier areas to provide shade and improve soil quality by fixing nitrogen for other tree crops. Traditionally, the genus has been prominently used for female infertility, gonorrhea [21], stomach pains, and microbial infections [22]. *Erythrina caffra *Thunb., the coast coral tree, native to Southeastern Africa, has warm red- to scarlet-coloured flowers which appear from the cold winter months up to spring. It is found in the coastal regions of the Eastern Cape and northern KwaZulu-Natal. In traditional medicine, its bark is used to treat sores, tuberculosis, respiratory infections, wounds, abscesses, arthritis and toothache. The infusions of the leaves are used as eardrops for earache. The decoctions of the roots are used for sprains [23, 24]. Despite its use suggesting antibacterial, anti-inflammatory, anti-blood-clotting, and analgesic effects, there is a dearth of scientific reports on *E. caffra *as a plant of medicinal importance. As a result, this study was aimed at investigating the ethnotherapeutic efficacies of *E. caffra* against bacteria associated with diarrhoea infection to justify its use in traditional medicine in the Eastern Cape of South Africa.

## 2. Materials and Methods

### 2.1. Collection of Plant Material

The bark materials of *E. caffra *were collected in September, 2010, from the plant growing within the University of Fort Hare campus in Alice, South Africa. The plant was authenticated in the Department of Botany, and a voucher specimen was prepared and deposited in the Griffin Herbarium of the University.

### 2.2. Extract Preparation

The bark sample was air-dried at room temperature, pulverized with a milling machine, and extracted in accordance with the description of Basri and Fan [25]. Briefly, about 100 g of the pulverized sample was extracted with 500 mL of ethanol for 72 h with shaking (Stuart Scientific Orbital Shaker, Staffordshire, UK). The extract was filtered through Whatman no. 1 filter paper and concentrated under reduced pressure at 40°C using a rotary evaporator (Laborota 4000-efficient, Heidolph city Germany). The crude extract collected was allowed to dry at room temperature to a constant weight of 3.5 g. The extract was redissolved in dimethylsulfoxide (DMSO) to the required concentrations for bioassay analysis. The reconstituted extract solution was sterilized by filtering through 0.45 *μ*m membrane filter and tested for sterility after membrane filtration by introducing 2 mL of the extract in 10 mL of sterile nutrient broth before being incubated at 37°C for 24 h. A sterile extract was indicated by the absence of turbidity in the broth after the incubation period.

### 2.3. Test Organisms and Inocula Preparation

The bacteria used in this study included *Proteus vulgaris* CSIR 0030, *Proteus vulgaris* KZN, *Shigella flexneri* KZN, *Micrococcus luteus*, *Enterococcus faecalis* KZN, *Staphylococcus aureus* OK_3_, *Shigella sonnei* (ATCC 29930), *Escherichia coli* (ATCC 25922), *Bacillus pumilus* (ATCC 14884), *Enterococcus faecalis* (ATCC 29212), *Pseudomonas aeruginosa* (ATCC 19582), and *Staphylococcus aureus* (ATCC 6538). The inocula of the test bacteria were prepared using the colony suspension method [26]. Colonies picked from 24 h old cultures grown on nutrient agar were used to make suspensions of the test organisms in saline solution to give an optical density of approximately 0.1 at 600 nm. The suspension was then diluted 1 : 100 by transferring 0.1 mL of the bacterial suspension to 9.9 mL of sterile nutrient broth before being used.

### 2.4. Antimicrobial Assay by Agar Diffusion (Inhibition Zones) and Agar Dilution Methods

For the initial determination of the antibacterial activity of the crude ethanol extract of *E. caffra*, the susceptibility screening of the test bacteria to the extract and ciprofloxacin, used as control, was determined by using the modified Kirby-Bauer diffusion technique [27] involving swabbing Mueller-Hinton agar (MHA) (Oxoid Ltd, Basingstoke, Hampshire, UK) plates with the resultant saline suspension of each adjusted bacterial strain. Wells were then bored into the agar medium using a heat-sterilized 6 mm cork borer. The wells were filled with 100 *μ*L of different concentrations of the extract (625 *μ*g/mL, 1250 *μ*g/mL, 2500 *μ*g/mL, 5000 *μ*g/mL, 10000 *μ*g/mL, and 20000 *μ*g/mL) and ciprofloxacin (2.5 *μ*g/mL) taking care not to allow spillage of the solutions onto the surface of the agar. The culture plates were allowed to stand on the laboratory bench for 1 h to allow proper diffusion of these solutions before being incubated at 37°C for 24 h. Wells in blank Mueller Hinton agar containing 10% DMSO representing the final concentration of the DMSO in the test plates without the extract served as negative control. The determinations were done in duplicates. After 24 h of incubation, the plates were examined if there is any inhibition zone. The diameters of the inhibition zones produced by each of the concentrations of the solutions were measured in millimeters [28] and interpreted using the CLSI zone diameter interpretative standards [29].

To determine the antibacterial activity of the extract by the agar dilution method described by Afolayan and Meyer [30], different concentrations of the extract ranging between 100 and 10000 *μ*g/mL were prepared in molten Mueller Hinton agar maintained in a water bath at 50°C and used for the agar dilution assay. One hundred microlitres (100 *μ*L) of the standardized bacterial cultures was aseptically dispensed and spread evenly on the agar plates. Two Mueller Hinton agar plates containing 5% ethanol representing the final ethanol concentration in the test plates without the extract served as negative controls. Another two blank plates containing only Mueller Hinton agar served as negative controls. Plates were incubated aerobically at 37°C for 24 h. Each test was done in triplicate, and any test agar plate lacking visible growth was considered the minimum inhibitory concentration of the extract.

### 2.5. Macrobroth Dilution for Determining Minimum Inhibitory Concentration (MIC)

Minimum inhibitory concentration (MIC) defined as the lowest concentration which resulted in maintenance or reduction of inoculums' viability was determined by serial tube dilution technique for the bacterial isolates. Different concentrations (19.5–10000) *μ*g/mL of the crude extract and (0.0195–10) *μ*g/mL of ciprofloxacin were differently prepared by serial dilutions in the Mueller Hinton broth medium. Each tube was then inoculated with 100 *μ*L of each of the adjusted bacterial strains. Two blank Mueller Hinton broth tubes, with and without bacterial inoculation, were used as the growth and sterility controls. The bacteria-containing tubes were incubated aerobically at 37°C for 24 h. After the incubation period, the tubes were observed for the MICs by checking the concentration of the first tube in the series (ascending extract and antibiotic concentrations) that showed no visible trace of growth. The first tube in the series with no visible growth after the incubation period was taken as the MIC.

### 2.6. Determination of Minimum Bactericidal Concentrations (MBC)

The MBC was determined by sampling all the macroscopically clear tubes and the first turbid tube in the series. Before being sampled, the tubes were gently mixed by flushing them with a sterile pipette, and a 100 *μ*L aliquot was removed. Each aliquot was placed on a single antibiotic-free nutrient agar plate in a single streak down the center of the plate in accordance with the method of Shanholtzer et al., [31]. The samples were allowed to be absorbed into the agar until the plate surface appeared dry (after 30 min). The aliquot was then spread over the plate by making a lawn of the bacterial culture with a sterile cotton swab. In many studies on microbial susceptibility, this subculturing method has been found satisfactory in eliminating the problem of antimicrobial agent carryover from the 100 *μ*L subcultured volume [32–35]. The growth and sterility controls were sampled in the same manner. The MBC-determining lawned plates were incubated for 24 h at 37°C. After the incubation periods, the lowest concentrations of the extract that did not produce any bacterial growth on the solid medium were regarded as the MBC values for this crude extract [36]. This observation was matched with the MIC test tube that did not show evidence of growth after 48 h of incubation.

### 2.7. Determination of Mechanisms of Antibiosis (Bactericidal or Bacteriostatic)

The mechanism of antibiosis of the extracts was calculated using the ratio of MBC/MIC or MIC_index_ as described by Shanmughapriya et al. [37] to elucidate whether the observed antibacterial effects were bactericidal or bacteriostatic. When the ratio of MBC/MIC was ≤2.0, the extract was considered bactericidal or otherwise bacteriostatic. If the ratio is ≥16.0, the extract was considered ineffective.

### 2.8. Determination of Rate of Kill

Assays for the rate of killing bacteria by the crude ethanolic extract were carried out using a modified plating technique of G. M. Eliopoulos and C. T. Eliopoulos [38] and Eliopoulos and Moellering [39]. The extract was incorporated into 10 mL Mueller Hinton broth in McCartney bottles at 1/2 MIC, 1 × MIC, and 2 × MIC. Two controls, one Mueller Hinton broth without extract inoculated with test organisms and Mueller Hinton broth incorporated with the extract at the test concentrations without the test organisms, were included. Inoculums density, approximately 10^5^ cfu/mL further verified by total viable count, was used to inoculate 10 mL volumes of both in the McCartney bottles and control bottles. The bottles were incubated at 37°C on an orbital shaker at 120 rpm. A 100 *μ*L aliquot was removed from the culture medium at 0, 4, and 8 h for the determination of cfu/mL by the plate count technique [40] by plating out 25 *μ*L of each of the dilutions. The problem of extract carryover was addressed by dilution as described previously by Pankuch et al., [41]. After incubating at 37°C for 24 h, emergent bacterial colonies were counted, cfu/mL calculated, and compared with the count of the culture control without the extract. 

## 3. Results 

The antibacterial activity of the stem bark ethanolic extract of *E. caffra* was evaluated by both agar diffusion and dilution assays against bacteria associated with diarrhoeal infection. The degree of the antibacterial activity was assayed by serial twofold dilution method to determine the minimum inhibitory concentration (MIC) of the extract [42]. The two assay methods showed that the bacteria exhibited varied susceptibility to the extract at the different concentrations used. The zones of inhibition obtained from 2.5 *μ*g/mL of ciprofloxacin ranged between 17 and 38 ± 1.0 mm. At the highest concentration of the extract (20000 *μ*g/mL), *Proteus vulgaris* CSIR 0030 had the widest zone of inhibition (23 ± 1.0 mm), while *Proteus vulgaris* KZN and *Staphylococcus aureus* (ATCC 6538) had the least zone of inhibition (15 ± 1.0 mm). Of these isolates, only *Proteus vulgaris* CSIR 0030, *Shigella sonnei* (ATCC 29930), *Escherichia coli* (ATCC 25922), *Enterococcus faecalis* (ATCC 29212), and *Pseudomonas aeruginosa* (ATCC 19582) had different zones of inhibition from 100 *μ*L of the least concentration (650 *μ*g/mL) used. The bacteria were not susceptible to 10% DMSO used in the control assay. The agar dilution indicated that the bacteria were susceptible to the extract at concentrations ranging between ≤100 and ≤1000 *μ*g/mL. From the agar dilution assay, *Micrococcus luteus*, *Proteus vulgaris* CSIR 0030, *Enterococcus faecalis* KZN, and *Staphylococcus aureus* OK_3_ had MICs less than or equal to 100 *μ*g/mL; *Shigella flexneri* KZN, *Proteus vulgaris* KZN, *Shigella sonnei* (ATCC 29930), *Bacillus pumilus* (ATCC 14884), *Enterococcus faecalis* (ATCC 29212), *Staphylococcus aureus* (ATCC 6538), and *Pseudomonas aeruginosa* (ATCC 19582) had their MICs less than or equal to 500 *μ*g/mL, while the susceptibility of *Escherichia coli* (ATCC 25922) indicated a higher MIC value less than or equal to 1000 *μ*g/mL (Table 1). 

The macrobroth dilution assay to determine the degree of the antibacterial activity showed that the minimum inhibitory concentrations (MICs) of the ciprofloxacin against the bacteria were generally below 1 *μ*g/mL, and the minimum bactericidal concentrations (MBCs) ranged between 0.0319 *μ*g/mL and 2.5 *μ*g/mL with the exception of *Staphylococcus aureus* OK_3_ having an MIC and MBC values of 1.25 *μ*g/mL. The MICs of the extract against all the bacteria isolates ranged between 39.1 and 625 *μ*g/mL, and the MBCs ranged between 78.1 and 625 *μ*g/mL. While the MICs of *Micrococcus luteus, Proteus vulgaris* CSIR 0030, *Enterococcus faecalis* KZN, and *Staphylococcus aureus* OK_3_ were less than 100 *μ*g/mL, those of *Shigella flexneri* KZN, *Proteus vulgaris* KZN, *Bacillus pumilus* (ATCC 14884), *Enterococcus faecalis* (ATCC 29212), and *Pseudomonas aeruginosa* (ATCC 19582) equal to 156.3 *μ*g/mL; *Shigella sonnei* (ATCC 29930), and *Staphylococcus aureus* (ATCC 6538) had 312.5 *μ*g/mL and *Escherichia coli* (ATCC 25922) had the highest MIC value of 625 *μ*g/mL (Table 2). The determination of the mechanisms of antibiosis indicated that both ciprofloxacin and the crude ethanolic extract were highly bactericidal against the entire test bacteria isolates, even though, the extract was considered not as effective as the antibiotic. While the MBCs of the antibiotic were similar or two- to four-folds higher than the MICs, the MBCs of the extract were similar or twofolds higher than the MICs. 

In the time-kill assay, the results presented in terms of the changes in the log⁡_10_ cfu/mL of viable colonies indicated that the extract exhibited a significant bactericidal activity. The bactericidal activity was defined as being equal to 3log⁡_10_  cfu/mL or greater reduction in the viable colony count relative to the initial inoculum [43]. The results of the time-kill assay are presented in Table 3. After 4 h of incubating the bacteria with the 1 × MICs and 2 × MICs, the average log⁡ reduction in the viable cell count ranged between −0.264log⁡_10_ and 0.916log⁡_10_ cfu/mL. After 8 h of incubation with these different concentrations, the average log⁡ reduction in the viable cell count ranged between −3.968log⁡_10_ and 1.105log⁡_10_ cfu/mL. At the 1 × MICs, incubating the bacteria for 4 h resulted in the reduction of the viable cell count ranging between 0.916log⁡_10_ and 1.851log⁡_10_ cfu/mL, while at 8 h, the reduction ranged between 0.183log⁡_10_ and 1.105log⁡_10_ cfu/mL. At 2 × MICs, incubating the bacteria for 4 h resulted in the reduction of the viable cell count that ranged between −0.264log⁡_10_ and 0.961log⁡_10_ cfu/mL while after 8 h of incubation, the average log⁡ reduction in the viable cell count ranged between −3.968log⁡_10_ and −0.425log⁡_10_ cfu/mL with *Micrococcus luteus*, *Proteus vulgaris* CSIR 0030, and *Staphylococcus aureus* OK_3_ being the most highly affected bacteria of all the isolates. 

## 4. Discussion

Medicinal plants have been unique sources of medicines and constituted the most common human use of biodiversity [44]. The traditional use of medicinal plants, being before the advent of antibiotics and other modern drugs [45] and their use worldwide for thousands of years with more than 80% of the world's population depending on traditional medicines for various diseases [46], implicated the anticipations of scientists that phytochemicals with adequate antibacterial efficacy will be useful for the treatment of bacterial infections [47]. Consequently, many ethnopharmacological studies are being conducted to determine their safety, efficiency, and discovery of new active principles in the recent times [48] while many medicinal plants and plants' products with antibiotic actions have become a significant source of many potent and powerful drugs [49, 50] as well as the main source of new leads for antimicrobial remedies and pharmaceutical development [51]. 

In this study, the crude ethanolic extract of *E. caffra* demonstrated significant inhibitory and bactericidal effects against all the test bacteria isolates. The differences in the values of MICs and MBCs suggested a selective antibacterial activity of the extract, while the varied susceptibility of the different bacteria isolates was extract concentration dependent. Although Ríos and Reco [52] suggested that MIC greater than 1000 *μ*g/mL of crude extracts or 100 *μ*g/mL for isolated compounds should be avoided and proposed that activity would be very interesting in MICs of 100 *μ*g/mL and 10 *μ*g/mL for crude extracts and isolated compounds respectively, Fabry et al. [53] defined active crude extracts as those having MIC values <8000 *μ*g/mL. Hence, since lower MIC and MBC values indicate higher efficacy [54], and phytochemicals are routinely classified as antimicrobials when susceptibility tests had MICs in the range of 100–1000 *μ*g/mL [55], the MICs and MBCs of less than 1000 *μ*g/mL, in this study, were considered to be of good activity. The MIC_index_ values, being less than or equal 2, indicated the bactericidal attribute of the crude ethanolic extract and suggested that bactericidal effects of the crude extract could be expected on most of the tested organisms [56] in a disease state. Where MIC equals MBC, the bactericidal potential with a broad spectrum and great therapeutic potential of the plant is indicated.

Furthermore, the resultant effect of incubating the bacteria at 2 × MICs was a significantly rapid reduction in the average log⁡ of the viable cells counted. This reduction is greater than the rate of kill observed in the bacteria isolates treated with the 1 × MICs. The significant reduction in the cell counts between 4 and 8 h of incubation period acknowledged the fact that the extract was highly bactericidal seeing that the bacterial colonies were almost totally wiped out after incubating for 8 h. On the contrary, there was a net growth of all the test isolates treated with the 1/2 × MICs of the extract. While the growth inhibition and efficacy of the crude ethanolic stem bark extract were dose and time dependent to present effective time-kill profiles for the tested bacteria, the results of the antibacterial assays determined by the agar diffusion and dilution methods as well as the macrobroth dilution assay are complementary. These results were further substantiated by the observed rate of kill and the effectiveness of the extract's bactericidal activity as indicated by the time-kill assay. While the degree of antibacterial activity may be accounted for by the flavonoids synthesized by plants in response to microbial infection [57], and the active components in the crude extract may be acting synergistically to produce good antibacterial effects [58], the disparity between the activities of the extract and the standard antimicrobial drug may be due to the mixtures of bioactive compounds present in the extract compared to the pure compound contained in the standard antibiotics [54]. 

Although *in vitro* tests do not necessarily confirm that plant extracts are either effective medicines or a suitable candidate for drug development, it provides a basic understanding of a plant efficacy and leads to the search for new active substances as well as validating its use in traditional medicine. In ethnotherapy, the recorded antibacterial activity of the crude ethanolic extract of *E. caffra* is of potential importance in the health care delivery system. It showed that the plant could be used as an alternative to orthodox antibiotics in the treatment of infectious diseases caused by these microorganisms. Since investigating the pharmacological activity of plant extract could afford the designing of less expensive therapeutic agents to be used in economically less privileged regions [59], the crude extract could offer a considerable potential opportunity for the development of new agents effective against infections currently difficult to treat [60]. With these expectations, many pharmaceutical companies have renewed their interest in investigating higher plants as sources for new lead structures and the development of standardized antimicrobial agents of proven efficacy, safety, and quality. Consequently, understanding the chemical nature and isolating the active principle(s) in *E. caffra* will provide an opportunity to synthesize new and effective antibacterial drugs. 

## 5. Conclusions 

The use of plants in the treatment of infections has been extensively applied by the local populace in South Africa. In this study, the great potential of *E. caffra* in the treatment of microbial infection such as diarrhoea was elucidated, while the bactericidal activity of the extract showed that the plant is of medicinal importance. To establish the therapeutic applicability of this plant in the treatment of microbial infections, investigation of its mechanisms of action, *in vivo* studies, and toxicological effects are ongoing in our research laboratory. A consideration of the degree of the bactericidal activity of the extract showed that there is a need for the isolation of the bioactive compounds. From this study, we concluded that the extract showed broader-spectrum antibacterial activity and can justify the use of *Erythrina caffra* in the folkloric medicine for treating gastrointestinal infections in South Africa.

## Figures and Tables

**Table 1:** Results of agar diffusion assays to determine the antibacterial activity of the crude ethanolic extract of *E. caffra. *

Tested bacterial isolates	Average zones of inhibition (±1.0 mm)							
	Agar diffusion assay							Agar dilution
	Ciprofloxacin	Ethanolic extract						
	2.5	650	1250	2500	5000	10000	20000	MIC
	*μ*g/mL							
*Micrococcus luteus *	17	0	0	0	14	17	19	≤100
*Shigella flexneri* KZN	26	0	0	13	15	15	17	≤500
*Proteus vulgaris* KZN	35	0	0	0	14	15	15	≤500
*Proteus vulgaris* CSIR 0030	31	14	17	18	20	21	23	≤100
*Enterococcus faecalis* KZN	19	0	0	13	15	16	19	≤100
*Staphylococcus aureus* OK_3_	38	0	0	12	14	17	19	≤100
*Shigella sonnei* (ATCC 29930)	22	13	14	15	17	18	20	≤500
*Bacillus pumilus* (ATCC 14884)	25	0	0	16	18	19	21	≤500
*Escherichia coli* (ATCC 25922)	26	11	12	13	15	17	19	≤1000
*Enterococcus faecalis* (ATCC 29212)	25	15	15	17	19	21	22	≤500
*Staphylococcus aureus* (ATCC 6538)	34	0	0	11	13	13	15	≤500
*Pseudomonas aeruginosa* (ATCC 19582)	26	13	13	15	17	18	21	≤500

**Table 2:** Antibacterial activity of the ethanolic extract of *Erythrina caffra *Thunb.

Tested bacterial isolates	Macrobroth dilution					
	Ciprofloxacin			Ethanolic extract		
	MIC	MBC	MIC_index_	MIC	MBC	MIC_index_
	μg/mL			μg/mL		
*Micrococcus luteus *	0.1563	0.3125	2	39.1	78.1	2
*Shigella flexneri* KZN	0.0390	0.0781	2	156.3	312.5	2
*Proteus vulgaris* KZN	0.3125	0.6250	2	156.3	156.3	1
*Proteus vulgaris* CSIR 0030	0.1563	0.1563	1	39.1	39.1	1
*Enterococcus faecalis* KZN	0.1563	0.6250	2	78.1	78.1	1
*Staphylococcus aureus* OK_3_	1.2500	1.2500	1	39.1	78.1	2
*Shigella sonnei* (ATCC 29930)	0.1563	0.1563	1	312.5	312.5	1
*Bacillus pumilus* (ATCC 14884)	0.0391	0.0319	1	156.3	156.3	1
*Escherichia coli* (ATCC 25922)	0.0195	0.0195	1	625.0	625.0	1
*Enterococcus faecalis* (ATCC 29212)	0.6250	2.5000	4	156.3	156.3	1
*Staphylococcus aureus* (ATCC 6538)	0.3125	0.6250	2	312.5	312.5	1
*Pseudomonas aeruginosa* (ATCC 19582)	0.0781	0.1563	1	156.3	312.5	2

**Table 3:** *In vitro* time-kill assessment of the crude ethanolic stem bark extract of *E. caffra*.

	Log_10_ Kill			Log_10_ Kill			Log_10_ Kill		
Tested bacterial isolates	(1/2) × MIC			1 × MIC			2 × MIC		
	0 h	4 h	8 h	0 h	4 h	8 h	0 h	4 h	8 h
*Micrococcus luteus *	2.187	2.895	3.233	2.207	1.135	0.183	2.244	−0.264	−2.213
*Shigella flexneri* KZN	2. 229	3.177	3.983	2.246	1.399	0.872	2.410	0.298	−1.526
*Proteus vulgaris* KZN	2.286	3.243	4.427	2.308	1.147	0.265	2.275	0.459	−1.503
*Proteus vulgaris* CSIR 0030	2.270	3.392	4.268	2.254	1.109	0.045	2.297	−0.161	−3.968
*Enterococcus faecalis* KZN	2.227	2.821	3.289	2.334	1.058	0.404	2.366	0.129	−1.741
*Staphylococcus aureus* OK_3_	3.177	4.384	5.986	2.889	0.916	0.381	3.238	−0.475	−2.686
*Shigella sonnei* (ATCC 29930)	2.453	3.243	4.276	2.553	1.172	0.878	2.683	0.961	−1.819
*Bacillus pumilus* (ATCC 14884)	2.442	3.772	4.478	2.398	1.413	0.697	2.778	0.448	−1.216
*Escherichia coli* (ATCC 25922)	2.135	3.327	5.482	2.247	1.548	0.899	2.541	0.678	−1.697
*Enterococcus faecalis *(ATCC 29212)	2.399	3.648	4.887	2.368	0.976	0.637	2.465	0.394	−0.425
*Staphylococcus aureus* (ATCC 6538)	2.387	3.512	4.674	2.351	1.391	0.902	2.391	0.681	−1.839
*Pseudomonas aeruginosa* (ATCC 19582)	2.724	3.805	4.921	2.923	1.851	1.105	2.612	0.831	−1.287

Key: −ve sign = extent of bacterial reductions as indicated by the Log (cfu/mL) at the respective sampling times.
